# Correlation between urine ACR and 24-h proteinuria in a real-world cohort of systemic AL amyloidosis patients

**DOI:** 10.1038/s41408-020-00391-2

**Published:** 2020-12-11

**Authors:** Alissa Visram, Abdullah S. Al Saleh, Harsh Parmar, Jennifer S. McDonald, John C. Lieske, Iuliana Vaxman, Eli Muchtar, Miriam Hobbs, Amie Fonder, Yi L. Hwa, Francis K. Buadi, David Dingli, Martha Q. Lacy, Angela Dispenzieri, Prashant Kapoor, Suzanne R. Hayman, Rahma Warsame, Taxiarchis V. Kourelis, Mustaqeem Siddiqui, Wilson I. Gonsalves, John A. Lust, Robert A. Kyle, S. Vincent Rajkumar, Morie A. Gertz, Shaji K. Kumar, Nelson Leung

**Affiliations:** 1 Division of Hematology, Mayo Clinic, Rochester, MI USA; 2 University of Ottawa, Ottawa Hospital Research Institute, Ottawa, ON Canada; 3 Department of Hematology, King Saud bin Abdulaziz University for Health Sciences, Riyadh, Saudi Arabia; 4 Division of Hematology, John Theurer Cancer Center at Hackensack University, Hackensack, NJ USA; 5 Department of Radiology, Mayo Clinic, Rochester, MI USA; 6 Department of Laboratory Medicine and Pathology, Mayo Clinic, Rochester, MI USA; 7 Institute of Hematology, Davidoff Cancer Center, Rabin Medical Center Petah- Tikvah, Petah Tikva, Israel; 8 Israel Sackler Faculty of Medicine Tel-Aviv University, Tel-Aviv, Israel; 9 Division of Nephrology, Mayo Clinic, Rochester, MI USA

**Keywords:** Haematological diseases, Signs and symptoms, Diagnosis

## Abstract

A 24-h urine protein collection (24hUP), the gold standard for measuring albuminuria in systemic AL amyloidosis, is cumbersome and inaccurate. We retrospectively reviewed 575 patients with systemic AL amyloidosis to assess the correlation between a urine albumin to creatinine ratio (uACR) and the 24hUP. The uACR correlated strongly with 24hUP at diagnosis (Pearson’s *r* = 0.87, 95% CI 0.83–0.90) and during the disease course (Pearson’s *r* = 0.88, 95% CI 0.86–0.90). A uACR ≥300 mg/g estimated a 24hUP ≥ 500 mg with a sensitivity of 92% and specificity of 97% (area under the receiver operating curve = 0.938, 95% CI 0.919–0.957). A uACR cutoff of 3600 mg/g best predicted a 24hUP > 5000 g (sensitivity 93%, specificity 94%), and renal stage at diagnosis was strongly concordant using either 24hUP or uACR as the proteinuria measure (k = 0.823, 95% CI 0.728–0.919). In patients with serial urine collections, a > 30% decrease in uACR predicted a > 30% decrease in 24hUP with a sensitivity of 94%. In conclusion, the uACR is a reliable and convenient method for ruling out proteinuria >500 mg per day, prognosticating renal outcomes, and assessing renal response to therapy. Further studies are needed to validate the uACR cutoffs proposed in this study.

## Introduction

Systemic light chain (AL) amyloidosis is a rare clinical entity, with an estimated incidence of 10–14 cases per million people in the United States^1,2^. The pathogenic immunoglobulin light chain in systemic AL amyloidosis is most often produced by a small clonal plasma cell population, and misfolded light chains aggregate and deposit in organs as amyloid fibrils, leading to organ toxicity and dysfunction^3^. Renal AL amyloid deposition predominantly involves the glomerulus, and therefore patients typically develop albuminuria, renal insufficiency, or nephrotic syndrome^4^. Renal involvement is seen in ~50–70% of systemic AL amyloid patients at diagnosis^5,6^, and is defined as 24-h urine protein (24hUP) >0.5 grams per day, that is predominantly albumin^7^. In patients with renal AL amyloidosis, the degree of renal insufficiency and proteinuria at diagnosis is predictive of the risk of progression to end stage renal disease (ESRD) requiring dialysis^8^. The reduction in proteinuria with plasma cell directed therapy has also been shown to correlate with prevention of end stage kidney disease and improved survival in systemic AL amyloidosis patients^8,9^. Therefore, the assessment of proteinuria in amyloidosis remains clinically relevant for prognostication at diagnosis, and for monitoring disease response.

Currently, the gold standard for assessment of proteinuria is a timed 24-hour urine collection that measures the total protein. However, a 24-hour protein (24hUP) collection is cumbersome for patients and is unreliable due to under or over collection and laboratory processing methods^10–14^. Therefore, the urine albumin to urine creatinine ratio (uACR) has been proposed as a convenient way to estimate 24hUP, and has been reported to be predictive in multiple diseases associated with albuminuria^15–18^. The uACR has also been shown to correlate with 24hUP when collected in systemic AL amyloidosis patients enrolled in clinical trials at diagnosis; however, this association has not been validated in a real-world setting^19^.

## Methods

We identified patients with systemic AL amyloidosis who were evaluated during their disease course at Mayo Clinic Rochester, between January 1, 2010 and September 30, 2019. Patients with a paired random spot urine (for uACR evaluation) and a 24hUP sample collected less than 7 days apart were included. Baseline characteristics and correlation analysis were performed only on the first paired samples available. Medical records were reviewed to verify that included patients had biopsy proven systemic AL amyloidosis. Patients on dialysis at the time of urine collection were excluded from this study. All patients had a serum creatinine tested within 1 day of random urine collection. At our center the Roche COBAS^®^ 6000 Analyzer is used to measure the urine albumin (Tina-quant Albumin Gen.2 reagent) and urine creatinine (CREP Gen.2 reagent) in order to obtain the uACR.

Demographic data, baseline disease characteristics, progression to dialysis, and follow-up dates were extracted through a chart review. The primary objective of this study was to assess the degree of correlation between uACR and 24hUP in systemic AL amyloid patients during the course of their disease. Our secondary objective was to validate the renal staging system for systemic AL patients using the uACR as a substitute for 24hUP. Lastly, we tested the relationship between uACR and a spot urine protein to creatinine ratio (uPCR) to a 24hUP collection. For this analysis we included systemic AL amyloidosis patients with both a urinalysis and random uACR collection on the same day, and a 24hUP collection within 7 days of the random urine collections. The protein to creatinine ratio was calculated using the total protein from the urinalysis, and the urine creatinine measurement from the uACR.

### Statistical analysis

Descriptive statistics were used to quantitate baseline characteristics. A Wilcoxon rank sum test was used to assess data that was not normally distributed. A Chi-square test was used to test categorical data. The correlation between spot uACR and 24hUP, and uPCR and 24hUP was assessed using the Pearson’s test. Linear regression analysis was used to construct a model to predict the 24hUP using uACR as the primary predictor, along with other variables that could affect the prediction of 24hUP (sex, body mass index, morning [AM] versus afternoon [PM] spot urine collection, serum creatinine at time of urine collection, and age at urine collection). A univariable analysis was conducted and significant variables (defined as a *p* value < 0.15) were included in a multivariable analysis. A final model to predict 24hUP was constructed based on the multivariable analysis.

Receiver operating characteristic (ROC) analysis was used to identify the best uACR cutoff to predict the following 24hUP cutoffs: ≥500 mg (to establish renal involvement^7,20^), ≥1000 mg (to establish progressive renal disease^7,20^), 24hUP > 5000 mg (for amyloid renal staging^8^), and <200 mg (as a reduction in proteinuria to this level has been associated with improved overall survival in amyloid patients). ROC curves were compared with a concordance statistic, using the Delong method. Cohen’s kappa statistic was used to assess the reliability of renal staging based on 24hUP versus uACR.

The Kaplan–Meier method was used to determine renal survival, defined as time from diagnosis to ESRD requiring dialysis. Patients who died without requiring dialysis were censored for the renal survival analysis. Univariable Cox proportional models were used to assess the hazard ratios and 95% confidence intervals for progression to dialysis. All statistical analyses were performed using JMP Pro v14.1 (SAS Institute, Cary, NC) and R (R Core Team, 2020). All *p* values were two sided and a level of <0.05 was considered significant. This study was approved by the Mayo Clinic Institutional Review Board.

## Results

A total of 575 patients with systemic AL amyloidosis had a paired 24hUP and uACR samples collected at Mayo Clinic within 7 days, and were included in this retrospective study. The median time between diagnosis and uACR collection was 24 months (IQR 0.9–77). Of the 575 patients, 155 had paired uACR and 24hUP samples collected within 30 days of diagnosis.

The median 24hUP at diagnosis was 2168 mg (interquartile range [IQR] 808–5795), with a median serum creatinine of 1.1 (IQR 0.9–1.5) mg/dL. Renal involvement at diagnosis was seen in 394 (69%) of included patients. At diagnosis, 54 out of 534 patients (10%) with a urine protein electrophoresis (UPEP) performed on a 24-hour urine collection had a monoclonal protein (MCP), and only 23 (4%) had >200 mg of urine MCP. Similarly, at the time of 24hUP collection 455 patients had a UPEP performed and 39 (9%) had a quantifiable MCP. The uACR and 24hUP samples were collected on the same day in 302 (52%) patients (median 0 days apart, IQR 0–1). At sample collection the median uACR was 211 (IQR 19–2516), and the median 24hUP was 447 (IQR 141–3059) mg. The median uACR varied significantly based on the timing of collection, with the median uACR from a morning collection being 123 (IQR 15–1604) mg/g compared to an afternoon collection median uACR of 596 (IQR 30–3947) mg/g (*p* < 0.001). The median follow-up from diagnosis was 58 months (IQR 22–107). Patient characteristics are summarized in Table 1.

**Table 1:** Baseline characteristics of systemic AL amyloidosis patients.

Characteristic	Systemic AL amyloidosis (*n* = 575)
Male—*n* (%)	370 (64)
Female—*n* (%)	205 (36)
Concomitant symptomatic multiple myeloma—*n* (%)	45 (8)
Amyloid light chain subtype	
Kappa—*n* (%)	169 (24)
Lambda—*n* (%)	406 (71)
Organ involvement at diagnosis	
Renal—*n* (%)	394 (69)
Cardiac—*n* (%)	306 (53)
Peripheral or autonomic neuropathy—*n* (%)	105 (18)
GI—*n* (%)	89% (15)
Liver—*n* (%)	66 (11)
At diagnosis	
Median 24 h urine proteinuria—mg (IQR)	2168 (808–5795)
Median urine monoclonal protein—mg (IQR)	0 (0–175)
Median serum creatinine—mg/dL (IQR)	1.1 (0.9–1.5)
Median dFLC at diagnosis—mg/L (IQR)^a^	188 (68–551)
dFLC < 10 mg/L—*n* (%)	28 (5)
dFLC > 1000 mg/L—*n* (%)	42 (7)
Bone marrow plasma cell burden—% (IQR)	10 (5–19)
At urine collection	
Age—years (IQR)	65 (59–71)
Median UACR—mg/g (IQR)	211 (19–2516)
Median spot urine albumin—mg/L (IQR)	170 (17–2129)
Median 24 h urine proteinuria—mg (IQR)	447 (141–3059)
Median eGFR—mL/min/1.73m^2^ (IQR)^b^	57 (34–74)
Median time between 24hUP and uACR—days (IQR)	0 (0–1)
Median time between diagnosis and uACR collection—months (IQR)	24 (0.9–77)
AM (before noon) UACR collection time—*n* (%)	346 (60)
PM (after noon) UACR collection time—*n* (%)	229 (40)
BMI—kg/m^2^ (IQR)	26.1 (23.4–29.7)

^a^*dFLC* difference in the involved to uninvolved free light chain.

^b^eGFR was calculated using the CKD-EPI formula, which incorporates age, sex, race, and serum creatinine^26^. Given our patient demographic, we assumed that all patients were non-African American.

Bold values indicate statistical significance.

### Correlating UACR with 24H protein

We explored the association between 24hUP and uACR in multiple subsets of our patients with systemic AL amyloidosis (subsets are characterized in Table 2). We found that uACR correlated equally well with the 24hUP in patients with samples within 30 days of diagnosis (*n* = 155, Pearson’s *r* = 0.87, 95% CI 0.83–0.90), or at any time in their disease course (*n* = 575, Pearson’s *r* = 0.88, 95% CI 0.86–0.90). The correlation remained strong even in patients with eGFR <30 mL/min/1.73 m^2^ (*n* = 127, Pearson’s *r* = 0.84, 95% CI 0.79–0.89) and proteinuria <3 g/day (*n* = 466, Pearson’s *r* = 0.81, 95% CI 0.77–0.84) at the time of urine sample collection.

**Table 2:** Summary of systemic AL amyloidosis patients (*n* = 575) included in subset analyses.

		Median (IQR) values at sample collection			
Description of subgroup	Number of patients	uACR (mg/g)	24hUP (mg)	dFLC (mg/L)	eGFR—(mL/min/1.73 m^2^)
Paired uACR and 24hUP samples <30 days from diagnosis^a^	155	1362 (87–4311)	1469 (286–5208)	242 (89–625)	62 (39–78)
Renal involvement at diagnosis	109	3329 (1149–5151)	3176 (1307–7823)	174 (70–491)	63 (38–78)
eGFR <30 mL/min/1.73 m^2^ at sample collection	127	2146 (334–5859)	2663 (654–5996)	31 (11–169)	18 (11–25)
24hUP < 3000 mg/day at sample collection	466	78 (12–743)	244 (124–1119)	27 (8–140)	59 (39–75)
≥2 paired uACR and 24hUP samples in patients with renal involvement at diagnosis^a^	224	959 (134–2560)^b^	1211 (279–3994)^b^	18 (5–72)^b^	52 (28–71)^b^
uACR, uPCR, and 24hUP available during disease follow-up	286	730 (33–3830)	1054 (180–4226)	61 (13–278)	51 (26–73)

^a^Paired uACR and 24hUP samples were collected a maximum of 7 days apart, and paired uPCR samples were included if a urinalysis was collected on the same day as the uACR.

^b^Laboratory values at the time of the first uACR collection are presented in this table.

In patients with systemic AL amyloidosis (*n* = 575), ROC analysis showed that a uACR cutoff of >280 mg/g was the best predictor of a 24hUP > 500 mg, with an area under the curve (AUC) of 0.988, sensitivity of 94%, and specificity of 97%. Table 3 outlines various uACR cutoffs correlating with 24UP values of interest. The correlation between paired uACR and 24hUP samples and uACR cutoff to predict a 24hUP > 500 mg/g was similar in patients with samples collected on the same day (*n* = 302, Pearson’s *r* = 0.87 [95% CI 0.84–0.89], uACR 294 mg/g with AUC 0.995) versus samples collected 1–7 days apart (*n* = 273, Pearson’s *r* = 0.89 [95% CI 0.86–0.91], uACR 283 mg/g with AUC 0.984). The ROC analysis was repeated in the subset of patients with samples available within 30 days of diagnosis, and the uACR cutoff that predicted a 24hUP > 500 mg was similar (*n* = 155, uACR 270 mg/g with AUC 0.992, sensitivity 98%, specificity 98%).

**Table 3:** ROC analysis using uACR to predict 24hUP in systemic AL amyloidosis patients (*n* = 575).

24hUP (mg) prediction threshold	Discriminant uACR (mg/g)	AUC (95% CI)	Sensitivity (%)	Specificity (%)
<200	131	0.938 (0.919–0.957)	95	82
>500	283	0.989 (0.983–0.995)	94	97
>1000	707	0.988 (0.982–0.995)	93	96
>5000	3580	0.976 (0.964–0.989)	94	94

A prior study reported that uACR >500 mg/g predicts 24hUP > 500 mg with the highest sensitivity and specificity, and so we assessed this cutoff in our patient cohort^19^. Based on our ROC analysis, a uACR cutoff of >500 mg/g predicted a 24hUP > 500 mg with a sensitivity of 86%, and a specificity of 99%. For simplicity, we used a uACR cutoff of 300 mg/g to predict a 24hUP of >500 mg (sensitivity 92%, specificity 97%). We found that 259 (97%) of patients with uACR >300 mg/g had a 24 h UP > 500 mg, and only 22 (8%) patients with uACR <300 mg/g had a 24hUP > 500 mg (*p* < 0.001). In the 155 patients with uACR and 24hUP samples available within 30 days of diagnosis, the definition of renal involvement (24hUP > 500 mg or uACR >300 mg/g) was concordant in 94% of cases (*p* < 0.001).

A simple linear regression model showed that a higher uACR was associated with a significantly higher 24hUP (β 1.03, 95% CI 0.99–1.06, R^2^ 0.775, *p* < 0.001)). A univariable linear regression was used to assess the effect of serum creatinine, time of collection (AM or PM), age at collection, sex, and body mass index (BMI) on the relationship between uACR and 24hUP. All of these variables were significant in the univariable analyses (*p* < 0.15) and were therefore included in the multivariable analysis. In the multivariable analysis sex, BMI, and age at uACR collection remained significant (Table 4). Variables significant in the multivariable linear regression were used to construct a model to predict 24hUP. A significant regression equation was found: E (24hUP_i_) = 372 + 1.04(uACR_i_) + 51(BMI_i_) − 23(age at uACR collection_i_) − 248(if male sex_i_). The 24hUP increased by 1.03 mg for every 1 mg/g increase in uACR, increased by 51 mg for each 1 kg/m^2^ increase in BMI, decreased by 23 mg for each 1 year increase in age, and males had a lower 24hUP by 248 mg. The overall model was statistically and clinically significant (*p* < 0.001 and adjusted R^2^ 0.795, respectively). Age at uACR collection, gender, and BMI did not confound the primary relationship between uACR and 24hUP, and no collinearity was observed.

**Table 4:** Linear regression analysis results.

	Univariable analysis	Multivariable analysis		
Variable	*P* value	Beta coefficient	95% CI	*P* value
uACR (mg/g)	**<0.001**	1.04	(0.99, 1.08)	**<0.001**
Sex (males)	**0.105**	−250	(−375, −125)	**<0.001**
BMI (kg/m^2^)	**0.015**	52	(30, 74)	**<0.001**
Age at uACR collection (years)	**0.122**	−22	(−35, −9)	**<0.001**
Time of collection (AM versus PM)	**<0.001**	67	(−58, 192)	0.295
Serum creatinine (mg/dL)	**0.001**	−49	(−102, 4)	0.072

Bold values indicate statistical significance.

We wanted to assess whether changes in the uACR predicted changes in the 24hUP, in order to assess if the uACR could be used to monitor fluctuations in proteinuria over time. Therefore, we studied the change in uACR and 24hUP at two timepoints, in 224 patients with renal amyloidosis at diagnosis that had collected at least two paired uACR and 24hUP samples, with the paired samples collected at least 30 days apart. The time from median diagnosis to first uACR collection (38.9 [IQR 2.8–82] months) was heterogeneous within this subgroup. Both paired samples were collected prior to first progression in 131(58%) of patients, of whom 40 had received an upfront autologous stem cell transplant, 73 received proteosome inhibitor based induction only, 14 received melphalan-based induction therapy, 2 received immunomodulatory drug induction, and 2 received a combination of PI and IMID induction therapy. Both serial samples were collected after initiation of second line therapy in 72 patients, and between first and second line therapy in 21 patients. The median time between the first and second uACR collection was 7 (IQR 3–13) months, and 147 (66%) still had a 24hUP ≥ 500 mg/g at the time of their first urine collection.

There was a strong correlation between the percent change in uACR and 24hUP (Pearson’s *r* = 0.841, 95% CI 0.798–0.876). Between the first and second uACR collection, the median change in eGFR was a decrease of 6.4% (IQR decrease of 18.6% to increase of 9.8%). Overall, between the two serial sample timepoints only 31 (14%) patients would have met renal progression criteria^8,21^ which assess progression based only on the change in eGFR.

The sensitivity and specificity of a decrease in uACR >30% correlating with a > 30% decrease in 24hUP was 94% and 87%, respectively. Similarly, a > 50% decrease in 24hUP correlated with a > 50% decrease in uACR with a sensitivity of 95% and a specificity of 88%. However, the sensitivity and specificity of a > 30% increase in 24hUP correlating with a > 30% increase in uACR was lower (67% and 82%, respectively). Similarly, a > 30% increase in 24hUP correlated with a > 30% increase in uACR with a sensitivity of 64% and a specificity of 89%.

### Evaluating the association between a protein to creatinine ratio and 24hUP

A total of 286 systemic AL amyloid patients had a urine protein to creatinine ratio (uPCR) and uACR collected on the same day, along with a 24hUP collection within 7 days of the spot urine collections. In 22 patients the random urine samples were collected within 30 days of diagnosis, and the median time from diagnosis of amyloidosis to random urine collection was 7(IQR 0–58) months. A urine MCP was quantifiable in 26 (9%) of patients at the paired 24hUP collection, and the median urine MCP was 139 (67–564) mg based on urine protein electrophoresis conducted using the 24 hour urine collection.

Seventy-three percent (*n* = 208) of patients included in this analysis had renal amyloid involvement at diagnosis. We found that in these patient samples, the uPCR correlated strongly with 24hUP (Pearson’s *r* = 0.83, 95% CI 0.80–0.87), as did the uACR (Pearson’s *r* = 0.88, 95% CI 0.85–0.90). However, in patients with lower levels of proteinuria (24hUP < 3000 mg), uACR correlated better with 24hUP (Pearson’s *r* = 0.81, 95% CI 0.77–0.86) compared to uPCR (Pearson’s *r* = 0.65, 95% CI 0.56–0.73).

We used ROC analysis to assess uACR and uPCR cutoffs that predicted significant proteinuria. In this patient cohort, the cutoffs that predicted >500 mg proteinuria in 24 h were a uACR of 324 mg/g (AUC 0.991, 95% CI 0.984–0.998, sensitivity 96%, specificity 99%) and uPCR 698 mg/g uPCR and 24hUP (AUC 0.991, 95% CI 0.983–0.999, sensitivity 95%, specificity 98%). When compared, the ROC curves of uACR versus uPCR to predict 24hUP > 500 mg were not significantly different (z = 0.03, *p* = 0.98).

The uPCR was collected in the AM in 150 (52%) patients, and in the PM in 136 (48%) of patients. The median uPCR was significantly lower in the AM versus the PM (540 versus 1651 mg/g, respectively, *p* < 0.001). Multivariable linear regression analysis showed that the timing of collection (AM vs PM) significantly affected the uPCR even after adjusting for other factors that could modify the effect or confound the relationship between uPCR and 24hUP (time of collection, age, serum creatinine, BMI). Given the significant variation in uPCR between AM and PM collections, we repeated the ROC analysis in AM and PM subgroups. We found the uPCR cutoff that best predicted a 24hUP > 500 mg was 563 mg/g (AUC 0.994) in the AM group, and 877 mg/g (AUC 0.992) in the PM group. Interestingly, there was less variation in the uACR cutoff that best predicted a 24hUP > 500 mg in the AM collection (312 mg/g, AUC 0.996) versus PM collection (324 mg/g, AUC 0.987).

### Validating renal staging for systemic AL amyloidosis using the uACR

To validate the renal staging system using uACR, we used a ROC analysis and determined that 24-hour proteinuria >5000 mg was predicted by a uACR of 3580 mg/g with the highest sensitivity and specificity (Table 3). For convenience we used uACR >3600 (sensitivity 93%, specificity 94%) as a substitute for 24hUP > 5000 mg in the renal staging model^8^. Of the 394 patients with renal involvement at diagnosis, 109 patients had a paired uACR and 24hUP collection within 30 days of diagnosis, and were therefore included in the renal staging validation analysis.

**Fig. 1:**
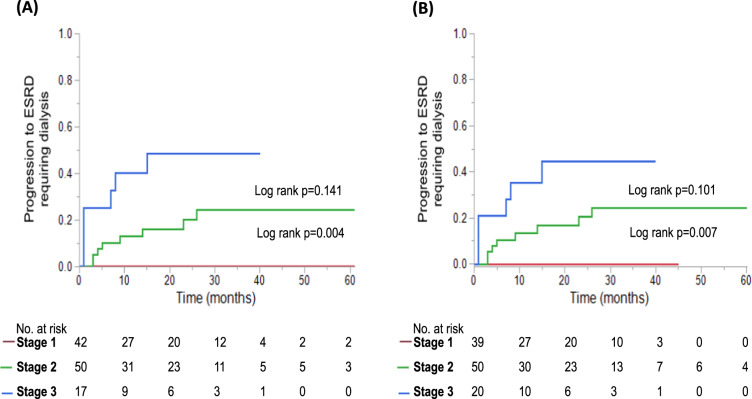
Renal staging at diagnosis using either uACR or 24hUP as the measure for proteinuria. The risk of progression to end stage renal disease (ESRD) requiring dialysis is estimated with the renal staging at diagnosis. Renal stages were applied using either **A** 24hUP > 5000 mg, or **B** uACR > 3600 mg/g as the measure of proteinuria. Stage 1 is indicated in red, stage 2 in green, stage 3 in blue.

The median time between diagnosis and uACR collection in the patients included in the renal staging analysis was 1 (IQR −1 to 17) day. The median follow-up duration from diagnosis was 19 months (IQR 3–31). Thirty-five (32%) of patients underwent an autologous stem cell transplant. At the time of uACR collection, the median eGFR was 62 (IQR 38–78) mL/min/1.73 m^2^, median serum creatinine was 1.1 (IQR 0.9–1.8) mg/dL, median uACR was 3329 (IQR 1149–5151) mg/g, and the median 24hUP was 3176 (IQR 1307–7823) mg. A total of 15 (14%) patients progressed to ESRD requiring dialysis, at a median of 5 (IQR 1–14) months from diagnosis. The renal staging system was applied using either 24hUP > 5000 mg or uACR >3600 mg/g as the criteria for proteinuria (Table 5 and Fig. 1). The stratification of patients by renal stage was strongly concordant using 24hUP and uACR (k = 0.823, 95% CI 0.728–0.919). All patients identified as stage 3 using 24hUP were also stage 3 using uACR. The risk of progression to ESRD requiring dialysis in stage 3 versus stage 2 patients was 3-fold higher using uACR (HR 2.9, 95% CI 1.04–8.33, *p* = 0.041) and 24hUP (HR 3.3, 95% CI 1.20–9.52, *p* = 0.021) as the marker of proteinuria. No patients with renal stage 1 using uACR or 24hUP progressed to dialysis during the course of this study’s follow-up.

**Table 5:** Renal staging system at time of urine collection (total *n* = 109).

	Renal staging (using 24hUP)	Renal staging (using UACR)
Stage		
1—n (%)	42 (40)	39 (36)
2—n (%)	50 (46)	50 (46)
3—n (%)	17 (16)	20 (18)
Dialysis rate at 2 years		
Stage 1—% (95% CI)	0 (0, 0)	0 (0, 0)
Stage 2—% (95% CI)	20 (7, 33)	20 (8, 32)
Stage 3—% (95% CI)	49 (22, 76)	45 (19, 71)

Stage 1: eGFR ≥ 50 mL/min/1.73 m^2^ and either 24hUP < 5000 mg or uACR < 3600 mg/g.

Stage 2: eGFR < 50 mL/min/1.73 m^2^ or one of 24hUP ≥ 5000 mg or uACR ≥ 3600 mg/g.

Stage 3: eGFR < 50 mL/min/1.73 m^2^ and either 24hUP ≥ 5000 mg or uACR ≥ 3600 mg/g.

eGFR was calculated using the CKD-EPI formula.

## Discussion

Given the cumbersome nature of 24hUP collections, both the uACR and the urine protein to creatinine ratio (uPCR) have been studied to estimate proteinuria in amyloidosis patients. This is the first large, real-world study showing that uACR correlates with strongly with a 24hUP in patients both at diagnosis and during follow-up. Although the uPCR was found to correlate with 24hUP in a small study of patients with various subtypes of amyloidosis, the Kidney Disease Improving Global Outcomes (KDIGO) organization guidelines recommend uACR over uPCR for estimating proteinuria given that uACR is more sensitive at detecting glomerular pathology especially at lower concentrations of albuminuria^13,22^. This was demonstrated in our study, as the correlation between uPCR and 24hUP decreased at lower concentrations of proteinuria. We also showed that the uPCR cutoffs to predict proteinuria >500 mg/day varied based on the time of sample collection, further limiting the utility of this test. In plasma cell dyscrasias such as amyloidosis, the uPCR may also be misleading because it does not differentiate albuminuria from monoclonal protein excretion. In systemic AL amyloidosis, assessing albuminuria is relevant for evaluating renal response to therapy. Therefore, in patients with concomitant urinary monoclonal protein excretion, which we would expect to be highest prior to therapy, it is possible that uPCR measurements may overestimate proteinuria. Although this could not be assessed in this study due to the small number of patients with a uPCR and uACR measurement at diagnosis, given this theoretical concern we advocate for the use of a uACR instead of a uPCR.

Palladini et al. have shown previously that uACR correlates well with 24hUP in systemic AL amyloid patients at diagnosis^19^. This study showed that the correlation between uACR and 24hUP remains strong throughout the disease course in patients with systemic AL amyloidosis, and is not affected by severely reduced GFR (defined as <30 mL/min/1.73 m^2^)^13,23^. We found that 24hUP could be accurately predicted with a linear regression equation using uACR as a predictor, and that timing of uACR collection (morning versus afternoon void) did not significantly affect this relationship. Importantly, in patients with renal amyloidosis we showed that the percentage change in uACR correlated well with the percent change in 24hUP within the same patients, therefore showing that uACR has limited intraindividual variation and can be used reliably to evaluate renal response to therapy.

In this study, we found that a uACR > 300 mg/g best predicted a 24hUP > 500 mg (sensitivity 92%, specificity 97%), the minimum threshold of proteinuria used to define renal involvement^7^. However, Palladini et al. found that a uACR >500 mg/g was the best predictor of a 24hUP > 500 mg (with a reported sensitivity 89% and specificity 97%). The discrepancy in the uACR thresholds between studies may be explained by variations in the test itself (e.g., differences in the laboratory measurements of urine creatinine), or the patient population. On average, males have more muscle mass than females and therefore excrete more urinary creatinine, leading to lower uACR values. Our study included a disproportionately higher number of men (64%) than women, which may have skewed our ROC analysis. In our dataset, a uACR cutoff of >300 mg/g was more sensitive than a uACR >500 mg/g at predicting a 24hUP > 500 mg, and was associated with a positive predictive value of 97% and negative predictive value of 94%. Our dataset comprised of known systemic AL amyloid patients, and so the prevalence of renal involvement was high. In the real-world setting, where systemic AL amyloidosis is a rare diagnosis with an estimated prevalence of 40.5 cases per million patients, the negative predictive value of a uACR <300 will be even higher; significant proteinuria (24hUP > 500 mg) can be ruled out in more than 93% of patients with a uACR <300 mg/g, making uACR an effective and convenient screen for renal involvement. The added value of a 24 hour urine collection at diagnosis in systemic AL amyloidosis is minimal, especially given the low rates of quantifiable urinary MCP in these patients. Furthermore, a > 30% decrease in uACR accurately predicted a > 30% decrease in 24hUP in 94% of patients, and therefore serial uACR assessments may be a reliable method to assess renal response. Given the high sensitivity of uACR at detecting renal response to therapy, and the convenience of this method for monitoring disease, incorporation of uACR into routine clinical practice may be warranted if these findings are validated in other studies. The sensitivity of a > 50% increase in uACR at predicting a > 50% increase in 24hUP was lower at 64%, therefore increases in uACR are not as reliable to assess for renal progression. However, it has been shown that patients with organ progression at the time of second line therapy have an inferior survival compared to those with hematologic progression alone, therefore suggesting that therapeutic interventions should be considered prior to organ progression^24,25^. Given the uACR does not have a high sensitivity at detecting organ progression, we would recommend that in those patients with hematologic progression, renal progression should be confirmed with a 24hUP. Our ability to assess disease response at standardized timepoints was limited by the heterogeneity in that the timing of serial paired uACR and 24hUP collections in relation to initiation of amyloid therapy.

Interestingly, the ROC cutoff for uACR that predicted 24hUP > 5000 mg with the highest sensitivity and specificity was 3600 mg, which was the same uACR cutoff that best predicted progression to dialysis at 6 months and was used to substitute for 24hUP > 5000 mg in the study by Palladini and colleagues^19^. We showed that even in our small sample size, renal staging was highly concordant when substituting uACR >3600 mg/g for 24hUP > 5000 mg, and that renal staging using uACR was predictive of progression to ESRD requiring dialysis.

This study has some inherent limitations due to its retrospective nature. At our center, patients receive instructions to submit a midstream urine sample within 30 min of collection to our laboratory, however compliance with these recommendations was not assessed. We were not able to control for comorbidities (e.g., diabetic nephropathy, hypertension, urinary tract infections), or preanalytical factors (e.g., recent exercise, upright posture) that could affect proteinuria measurements. Due to the intraindividual variability in uACR measurements, the KDIGO guidelines recommend that at least two uACR samples should be collected at least 1 week apart in order to diagnose persistent albuminuria^13^. It would be useful to assess whether a second confirmatory uACR test improves the ability of a uACR to predict proteinuria. Furthermore, in this study the 24hUP was used as the gold standard comparator, even though 24hUP samples are often collected inaccurately^10,11^. The urinary creatinine excretion remains relatively stable within patients from day to day, and the adequacy of urine collection can be evaluated by comparing the predicted and measured urinary creatinine concentrations in a 24hUP collection. Therefore, the correlation between uACR and 24hUP may have been stronger if the urinary creatinine was used as a measure to ensure only accurate 24hUP collections were used in the analysis.

In conclusion, this study showed that in systemic AL amyloidosis patients uACR correlates strongly with 24hUP and can be reliably used to rule out significant proteinuria at diagnosis, and monitor changes in proteinuria during follow-up. The uACR can also be used as a measure of proteinuria in the renal staging system, in order to prognosticate the risk of progression to dialysis. We believe that uACR is a convenient and reliable method of ruling out renal involvement at diagnosis, prognosticating the risk of progression to ESRD in those with renal involvement, and monitoring for renal response to therapy in patients with systemic AL amyloidosis. However, further studies are need to validate the uACR cutoffs proposed in this study and assess the utility of serial uACR measurements in assessing renal response to therapy.
